# *Vellozia flavicans* Mart. ex Schult. hydroalcoholic extract inhibits the neuromuscular blockade induced by *Bothrops jararacussu* venom

**DOI:** 10.1186/1472-6882-14-48

**Published:** 2014-02-08

**Authors:** Natália Tribuiani, Alexandro Mateus da Silva, Miriéle Cristina Ferraz, Magali Glauzer Silva, Ana Paula Guerreiro Bentes, Talita Signoreti Graziano, Marcio Galdino dos Santos, José Carlos Cogo, Eliana Aparecida Varanda, Francisco Carlos Groppo, Karina Cogo, Yoko Oshima-Franco

**Affiliations:** 1 Pharmaceutical Sciences post-graduation program, University of Sorocaba (UNISO), Rodovia Raposo Tavares km 92.5, Sorocaba, SP CEP 18023-000, Brazil; 2 Piracicaba Dental School – University of Campinas (UNICAMP), Av. Limeira 901, Piracicaba, SP CEP 13414-903, Brazil; 3 Environmental Sciences post-graduation program, PGCiamb, Tocantins Federal University (UFT), Av NS 15 ALC NO 14, 109 Norte, Porto Nacional, Tocantins CEP 77001-090, Brazil; 4 Serpentárium of the Vale do Paraíba University (CEN - UNIVAP), Av Shishima Hifumi 2911, São José dos Campos, SP CEP 12244-000, Brazil; 5 Pharmaceutical Sciences Faculty of Araraquara, São Paulo State University (UNESP), Rodovia Araraquara-Jau, Km 1, Araraquara, São Paulo CEP 14801-902, Brazil; 6 Dentistry Department, de Santo Amaro University (UNISA), R. Prof. Eneas de Siqueira Neto 340, Santo Amaro, SP CEP 04829-300, Brazil

**Keywords:** Antimicrobial, Antiophidian, Medicinal plant, Snake venom

## Abstract

**Background:**

Snakebite is a significant public health issue in tropical countries. In Brazil, some of the most common snake envenomations are from *Bothrops. Bothrops* bites trigger local and systemic effects including edema, pain, erythema, cyanosis, infections, and necrosis. *Vellozia flavicans* is a plant from the Brazilian “cerrado” (savanna) that is popularly used as an anti-inflammatory medicine. Since inflammation develops quickly after *Bothrops* bites, which can lead to infection, the aim of the present study was to observe possible anti-snake venom and antimicrobial activities of *V. flavicans* (Vf).

**Methods:**

The chromatographic profile of the main constituents from the Vf leaf hydroalcoholic extract was obtained by thin-layer chromatography (TLC). The anti-snake venom activity was measured by Vf’s ability to neutralize the *in vitro* neuromuscular blockade caused by *Bothrops jararacussu* venom (Bjssu) in a mouse phrenic nerve-diaphragm model (PND). After a 20 min incubation, preparations of PND were added to Tyrode’s solution (control); Vf (0.2, 0.5, 1, and 2 mg/mL); 40 μg/mL Bjssu; pre-incubation for 30 min with Bjssu and 1 mg/mL Vf; and a Bjssu pretreated preparation (for 10 min) followed by 1 mg/mL Vf. Myographic recording was performed, and the contractile responses were recorded. The antimicrobial activity (minimum inhibitory concentration [MIC] and minimum bactericidal concentration [MBC]) was obtained for *Staphylococcus aureus*, *Pseudomonas aeruginosa*, *Escherichia coli*, and *Enterococcus faecalis*, using gentamicin and vancomycin as positive controls.

**Results:**

TLC analysis yielded several compounds from Vf, such as flavonoids (quercetin) and phenolic acids (chlorogenic acid). Bjssu completely blocked the contractile responses of PND preparations, while Vf preserved 97% (±10%) of the contractile responses when incubated with Bjssu. In the PND pretreated with Bjssu, Vf was able to inhibit the neuromuscular blockade progress. MIC and MBC of Vf ranged from 2.5 to 5.0 mg/mL for *P. aeruginosa* and *S. aureus* strains, while no antimicrobial activity was observed for *E. coli* and *E. faecalis*.

**Conclusions:**

The hydroalcoholic extract from Vf leaves was able to neutralize and decrease the *in vitro* neuromuscular blockade caused by Bjssu. However, it did not show significant antimicrobial activity against the tested bacteria.

## Background

Envenomation from snake attacks is a significant public health problem in rural areas of tropical and subtropical countries. According to the World Health Organization, there are at least 421,000 accidents involving snakes and 20,000 deaths worldwide from snakebite each year [1].

In Brazil, the genus *Bothrops* is a major group of snakes that commonly cause envenomation. Snakebites from this genus, which includes those of *Bothrops jararacussu*, are characterized by local and systemic effects, such as inflammation at the bite site, ecchymosis, bleeding, and skin infection with abscess. Snakebites are usually associated with inoculation of bacteria present in the snake’s mouth, which can lead to necrosis, gangrene, and amputation [2].

There are few reports on the *in vivo* neurotoxicity induced by *B. jararacussu* venom. Some reports detail unspecified signs [3-5] or describe systemic effects including blindness, blurred vision, difficulty in swallowing, and paralysis, which are reminiscent of the actions of *Crotalus* venom [6]. However, the *in vitro* irreversible neuromuscular blockade induced by *B. jararacussu* venom, which was first demonstrated by Rodrigues-Simioni et al. [7], has inspired other studies with other species of *Bothrops*.

*Vellozia flavicans* Mart. ex Schult. (Velloziaceae) is a native plant from the Brazilian “cerrado” (savanna) vegetation, popularly known as “canela-de-ema”. It is an herbaceous-shrub used as an anti-inflammatory and anti-rheumatic in folk medicine [8]. The Velloziaceae family contains about 270 species of tropical monocotyledonous plants, many of which are found in the Brazilian tropical scrub growing on rock outcrops [9]. Branco et al. [10] characterized the chemical constituents of *V. graminifolia*, which shares phytochemical groups already found in other *Vellozia* plants, such as flavonoids [11,12], diterpenoids [13-15], and triterpenoids [16,17]. Although the phytochemical elucidation is relatively advanced for this family, little is known about the pharmacological properties of specific species.

Plants used as anti-inflammatory medicines could be potentially effective for treating snakebites. This pharmacological relation has been confirmed by studies that found both anti-inflammatory and anti-venom properties from some plant extracts and their compounds [18,19]. Therefore, as *V. flavicans* is popularly used as anti-inflammatory medicine, it could have also some anti-venom activity. To test this hypothesis, a model using *B. jararacussu* venom is better than other venoms, such as *Crotalus durissus terrificus*, because the latter does not induce significant inflammation in the tissues around the snake bite [20]. *B. jararacussu* venom causes inflammation at the bite site and induces an irreversible neuromuscular blockade *in vitro*, but not *in vivo*[7].

The aim of the present study was to observe the ability of a hydroalcoholic extract obtained from *V. flavicans* leaves to neutralize the *in vitro* neuromuscular blockade caused by *B. jararacussu* venom in a mouse phrenic nerve-diaphragm model. In addition, the antimicrobial activity of the same extract was verified against *Staphylococcus aureus*, *Pseudomonas aeruginosa*, *Escherichia coli*, and *Enterococcus faecalis* strains, using gentamicin and vancomycin as positive controls. Thin-layer chromatography (TLC) was used to observe the main chemical constituents of the extract.

## Methods

### Plant material

The leaves of *V. flavicans* were collected at Porto Nacional, Tocantins State, Brazil, in September 2011, at S10° 44' 08.3" and W048° 21' 46.7". The plant was identified by the Botany Section from Biology Department of Tocantins Federal University, where a voucher specimen was deposited (HTO 8533). The *V. flavicans* leaves were dried at 37°C over 7 days and then powdered, ground in a mill, and macerated over 5 days in 70% ethanol. Then, the suspension was percolated (under protection against light) at 20 drops/min, resulting in a 10% (w/v) hydroalcoholic extract [21]. The extract was concentrated under reduced pressure, lyophilized, and stored at room temperature without light and humidity until the assays were performed.

### Thin-layer chromatography

Aliquots of *V. flavicans* extract (10% w/v solved in ethanol 70% and sonicated for 20 min) were spotted (5 times) on thin-layer silica gel plates (0.3 mm thick, Merck, Germany) and compared with a collection of reference phytochemicals [22]. The solvent system consisted of ethylacetate:formic acid:acetic acid:water (100:11:11:24, w/v). Both ethyl acetate and acetic acid were provided by Chemco Ind. (Campinas, SP, Brazil), while formic acid was supplied by Synth Chemical Co. (São Paulo, SP, Brazil). The standard phytochemical groups were flavonoids (quercetin and rutin) and phenolic acids (caffeic and chlorogenic acids), suspended in 1% methanol (w/v, P.A. solution, Sigma Chemical Co., St. Louis, MO, USA). The separated spots were visualized (under UV light at 360 nm) with NP/PEG as follows: 5% (v/v) ethanolic NP (diphenylboric acid 2-aminoethyl ester, Sigma Chemical Co., St. Louis, MO, USA) followed by 5% (v/v) ethanolic PEG 4000 (polyethylene glycol 4000, Synth Chemical Co., São Paulo, SP, Brazil). The retention factors (Rf) of the extract spots were compared with the Rf of standards.

### Antiophidian activity

#### V. flavicans hydroalcoholic extract solubilization

The lyophilized extract was solubilized in 30 μL dimethyl sulfoxide (DMSO; Sigma) and added to Tyrode’s solution (5 mL) before addition to the bath containing the biological preparation (see below). This DMSO concentration did not cause changes in the basal response of the neuromuscular preparation [23].

#### Venom

Crude venoms were collected from male and female adult *B. jararacussu* specimens (Bjssu, DL50_mouse, e.v._ = 5.18 mg/kg), in a glass receptacle covered with plastic paraffin film to avoid saliva contamination. The snake specimens are kept in the Serpentário do Centro de Estudos da Natureza – CEN at the University of Vale do Paraiba (UNIVAP, São José dos Campos, SP, Brazil) [24]. After collection, the venom was immediately placed in an ice bath, lyophilized, and kept in a refrigerator at 4°C. This process was certified by Professor Dr José Carlos Cogo from UNIVAP.

#### Animals

Male Swiss white mice (26–32 g) were provided by Animais de Laboratorio (Anilab, Paulinia, Brazil) and housed at 25 ± 3°C on a 12 h light/dark cycle, with food and water *ad libitum*. This study was approved by the institutional Commission for Ethics in the Use of Animals (CEUA) at the Federal University of São Carlos (UFSCAR) under protocol number 063/2012. All the experiments were performed following the guidelines of the Brazilian Society of Laboratory Animal Science (SBCAL).

#### Mouse phrenic nerve-diaphragm muscle (PND) preparation

The phrenic nerve-diaphragm [25] was obtained from mice anesthetized with halothane and killed by exsanguination. The diaphragm was removed and mounted under a tension of 5 g/cm in a 5 mL organ bath containing aerated Tyrode’s solution (control). This solution was composed of the following constituents (in mM): NaCl 137, KCl 2.7, CaCl_2_ 1.8, MgCl_2_ 0.49, NaH_2_PO_4_ 0.42, NaHCO_3_ 11.9, and glucose 11.1.

After stabilization with 95% O_2_ / 5% CO_2_ (v/v), the pH of the solution was 7.0. Preparations were indirectly stimulated with supramaximal stimuli (4× threshold, 0.06 Hz, 0.2 ms) delivered by bipolar electrodes from a stimulator (model ESF-15D, Ribeirão Preto, SP, Brazil) to the nerve. Isometric twitch tension was recorded with a force displacement transducer (cat. 7003, Ugo Basile, Italy) coupled to a 2-Channel Recorder Gemini physiograph device (cat. 7070, Ugo Basile) via a Basic Preamplifier (cat. 7080, Ugo Basile). The PND myographic recording was performed according to Ferraz et al. [26]. PND was allowed to stabilize for at least 20 min before addition of the following treatments: 1) Tyrode’s solution (control, n = 6); 2) lyophilized *V. flavicans* extract (0.2, 0.5, 1, and 2 mg/mL, n = 6); 3) 40 μg/mL Bjssu venom (n = 6); 4) pre-incubation for 30 min of 40 μg/mL Bjssu venom and 1 mg/mL *V. flavicans* extract (n = 6), and 40 μg/mL Bjssu venom pretreated preparation (10 min) followed by 1 mg/mL *V. flavicans* extract (n = 6). The venom concentration was based on a dose previously described in an irreversible *in vitro* neuromuscular blockade assay [27].

### Antimicrobial activity

#### Bacterial strains and culture conditions

The following bacterial strains were purchased from American Type Culture Collection (ATCC): *Escherichia coli* ATCC 25922, *E. coli* ATCC 10536, *Enterococcus faecalis* ATCC 29212, *Pseudomonas aeruginosa* ATCC 25619, *P. aeruginosa* ATCC 27853, *Staphylococcus aureus* ATCC 6538, *S. aureus* ATCC 14458, *S. aureus* ATCC 29213, methicillin-resistant *S. aureus* (MRSA) ATCC 33591, and methicillin/oxacillin resistant *S. aureus* (MRSA/ORSA) ATCC 43300. The cultures were stored at -80°C in Tryptic soy broth (TSB; Difco Laboratories, Detroit, MI, USA) containing 40% (v/v) glycerol and were routinely cultured in Tryptic soy agar (TSA; Difco Laboratories) under aerobic conditions at 37°C.

#### Determination of minimum inhibitory (MIC) and bactericidal (MBC) concentrations

MIC and MBC values were determined by using the broth microdilution method according to the guidelines from the Clinical and Laboratory Standards Institute (CLSI) protocol M07-A9. Briefly, four to five colonies were harvested from pure cultures growing on TSA and were used to prepare a bacterial inoculum. Colonies were transferred into tubes containing 5 mL of TSB and cultured at 37°C until reaching turbidity equivalent to a 0.5 McFarland standard (approximately 1.5 × 10^8^ CFU/mL).

*V. flavicans* stock solutions in 50% ethanol (Merck, Darmstadt, Germany) were diluted (2-fold serial dilutions) in 100 μL Mueller Hinton Broth (MHB, Difco Laboratories) in 96-well polystyrene microtiter plates at concentrations ranging from 0.004 mg/mL to 10 mg/mL.

The bacterial inoculum (100 μL) was then added into each well, allowing a final concentration of 5 × 10^5^ CFU/mL. Plates were incubated under aerobic conditions at 37°C for 18 h and visually inspected for turbidity after incubation. MIC was recorded as the lowest extract concentration that caused no turbidity. Solvent controls were assayed with the treatments to verify their possible antibacterial activity. Results were also compared with vancomycin, gentamicin (positive controls), and ethanol (negative control).

For MBC determination, 10 μL from each well with no visible growth were plated on TSA and incubated at 37°C for 24 h. MBC was considered as the lowest extract concentration that completely inhibited (100%) the bacterial growth. Both MIC and MBC values (mg/mL) were averaged from two different experiments performed in replicate.

### Statistical analysis

Results are shown as the mean ± SEM. Number of experiments (n) is indicated in the legend of the figure. Student’s *t*-test was used to compare all data and the significance level was set at 5%.

## Results and discussion

One of the criteria for selecting plants for pharmacological studies is their traditional use in folk medicine (ethnopharmacology). Selection is also based on chemical composition, especially considering certain compounds with well-known pharmacological activity [28,29]. In the present study both criteria were considered, since *V. flavicans* is traditionally used as an anti-inflammatory and antirheumatic. In addition, its chemical composition has been studied. Velloziaceae mainly contain flavonoids (phenolic compounds), diterpenes, and triterpenoids as the main secondary metabolites. Terpenoid is a term used to indicate that the substances have a common biosynthetic origin, the isoprene molecule. Flavonols are related to natural resistance factors and have some biological effects including antimicrobial and cardiovascular activities [30]. Terpenoids have other functions including growth-regulating properties, communication, and defense against insects [22].

**Figure 1:**
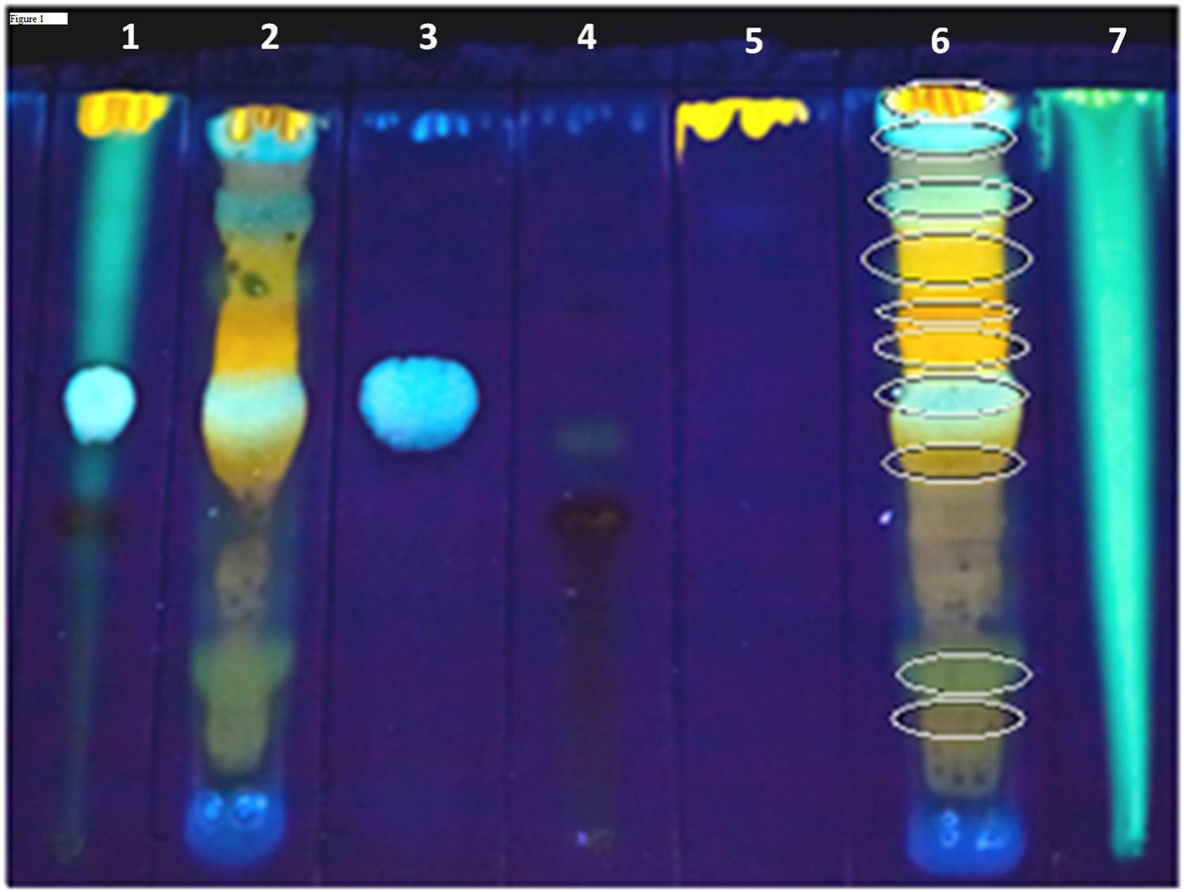
**Thin Layer Chromatography performed by using ethyl acetate:formic acid:acetic acid:water (100:11:11:24) as solvents.** Developer: NP/PEG. Phytochemical standards (inside circles): 1 – mixture of commercial phytochemicals (chlorogenic acid, rutin, quercetin caffeic acid); 2 – *V. flavicans* extract; 3 – chlorogenic acid (Rf = 0.60); 4 – rutin (Rf = 0.48); 5 – quercetin (Rf = 0.99); 6 - *V. flavicans* extract, showing Rf values of 0.99, 0.89, 0.80, 0.74, 0.70, 0.66, 0.60, 0.49, 0.23 and 0.13; 7 – caffeic acid (Rf = dragged spot). *V. flavicans* extract spots are suggestive of several flavonoids (yellow/orange fluorescence) and phenolics constituents (blue fluorescence), including quercetin and chlorogenic acid, respectively. Rf = retention factor.

After collection, botanical identification, stabilization, grinding, and extraction, the plant extract was qualitatively analyzed by TLC [31] to obtain a chromatographic fingerprint. TLC provides evidence of the plant’s chemical composition. Figure 1 shows the TLC profile of *V. flavicans* hydroalcoholic extract. Spots 2 and 6 indicate phenolic compounds (observe the highlighted area, Rf values were 0.99, 0.89, 0.80, 0.74, 0.70, 0.66, 0.60, 0.49, 0.23, and 0.13) including flavonols (yellow to orange fluorescence) and phenolic acids (blue fluorescence). A mixture of commercial phytochemicals (chlorogenic acid + rutin + quercetin + caffeic acid) is visualized in spot 1, chlorogenic acid in 3, rutin in 4, quercetin in 5, and caffeic acid in 7. Among the many compounds, note the presence of chlorogenic acid (Rf 0.60) and quercetin (Rf 0.99) in *V. flavicans* extract, representing phenolic acids and flavonols, respectively. All of these phytochemicals were previously reported in Velloziaceae [10-17].

Chlorogenic and caffeic acids may act as antidotes against snake venoms by binding to proteins through hydrophobic interactions and hydrogen bonds [32]. Quercetin is as a potent inhibitor of lipoxygenase, which explains its anti-inflammatory properties. Quercetin has a phenolic hydroxyl group on carbon 5, a pyronic carbonyl in close proximity, and coplanarity [33].

Even using other solvent systems, terpenoids were not found when compared with their representative β-sitosterol, the most abundant of the phytosteroids. Thus, the TLC profile, acting as a plant quality control, allowed the characterization of *V. flavicans* extract and comparison with literature data. However, the pharmacological activity is mainly attributed to flavonols.

Mors et al. [33] proposed a correlation between the ability of plants and their chemical components to neutralize snake venoms, and their anti-inflammatory and antihepatotoxic properties. In the present study, *B. jararacussu* venom was chosen to test if a plant with anti-inflammatory properties also has antiophidian potential, because *B. jararacussu* venom causes inflammation at the bite site [34] and causes an irreversible neuromuscular blockade *in vitro*[7,35,36].

The clinical effects of *B. jararacussu* venom are well described by Milani-Júnior et al. [37] and are similar to those of other *Bothrops* species. Although the mechanism of cytokine production induced by *Bothrops* venoms is not completely understood [38], the compounds leading to inflammation are well established. Therefore, since the anti-inflammatory properties are already attributed to *V. flavicans*[8], we decided to test the ability of this plant extract in neutralize the envenomation caused by *B. jararacussu* venom in a well-controlled and previously validated experimental design.

The World Health Organization has included snakebite envenoming as a neglected disease [39]. Few efforts have been carried out to change this, even considering the existing serum therapy. As observed in the present study and some other studies, plants could be an alternative or a complementary therapy to serum treatment.

**Figure 2:**
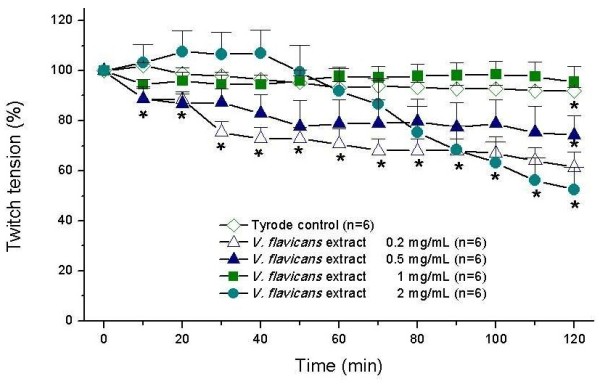
**Mouse phrenic nerve-diaphragm preparations (indirect stimuli). Concentration-response curve of *V. flavicans *extract.** Each point represents mean ± SEM. * = statistically significant differences (p < 0.05) in comparison with Tyrode control.

Figure 2 shows the dose-response curve (in mg/mL) of *V. flavicans* hydroalcoholic extract. Concentrations of 0.2 mg/mL (p < 0.05); 0.5 mg/mL (p < 0.05) and 2 mg/mL (p < 0.05), but not 1 mg/mL (p > 0.05), showed significantly less twitch tension than Tyrode’s control solution, indicating that these concentrations were able to interfere with the normal contraction of PND*.* A better result was obtained with 1 mg/mL *V. flavicans* extract, since this concentration did not change the basal response of neuromuscular preparation. Curiously, the low concentrations (0.2 and 0.5 mg/mL) caused a decrease in the baseline (Tyrode’s solution). This result suggests a parallel regulation on skeletal muscle excitability by an unclear mechanism, since other endogenous signaling pathways are capable to compensate the cholinergic control of movement. Glutamate, for example, was recently suggested as a co-transmitter of acetylcholine in motoneurons of mammalian neuromuscular junction [40,41].

**Figure 3:**
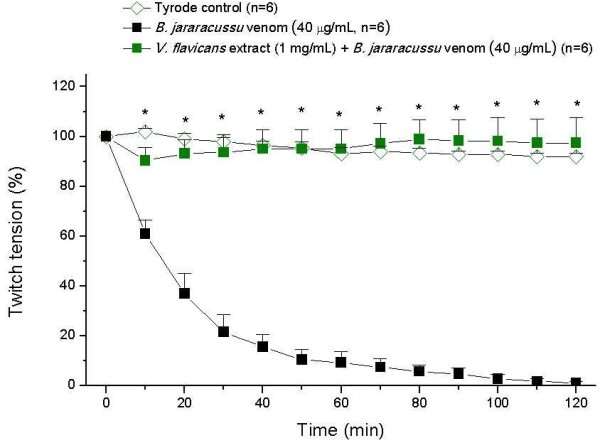
**Isolated mouse phrenic nerve-diaphragm preparations (indirect stimuli).** Each point represents mean ± SEM. * = statistically significant differences (p < 0.05) in comparison with the venom.

Figure 3 shows the pharmacological profile of preincubated (30 min) venom (40 μg/mL) plus *V. flavicans* extract (1 mg/mL) compared with the venom alone and Tyrode’s solution. The extract was able to completely abolish the decreased twitch tension caused by *B. jararacussu* venom at all times. At the end of experiment (120 min), the preparation with *V. flavicans* extract was 97% (±10%) responsive, indicating extract efficacy.

Effectiveness of mixing a plant extract or an isolated phytochemical with a venom to abolish the venom toxic effects is not guaranteed. Success depends on the venom and plant constituents. The main constituents of *V. flavicans* very closely reproduce the results obtained with the hydroalcoholic extract of *Casearia sylvestris* Sw. [42] or *Casearia gossypiosperma* Briq. [43], which are rich in flavonoids and polyphenols. However, the results were not similar with those observed for *Dipteryx alata*, which is rich in triterpenoids [26,44]; *Mikania laevigata*, which is rich in coumarin [45]*; Plathymenia reticulata* Benth., which is rich in tannins [46]; or *Camellia sinensis*, which is rich in catechins [36]. Nevertheless, except for tannins and tannic acid, which act by a clearly visible precipitation [45], most of the mechanisms of action of other phytochemicals remain unclear.

**Figure 4:**
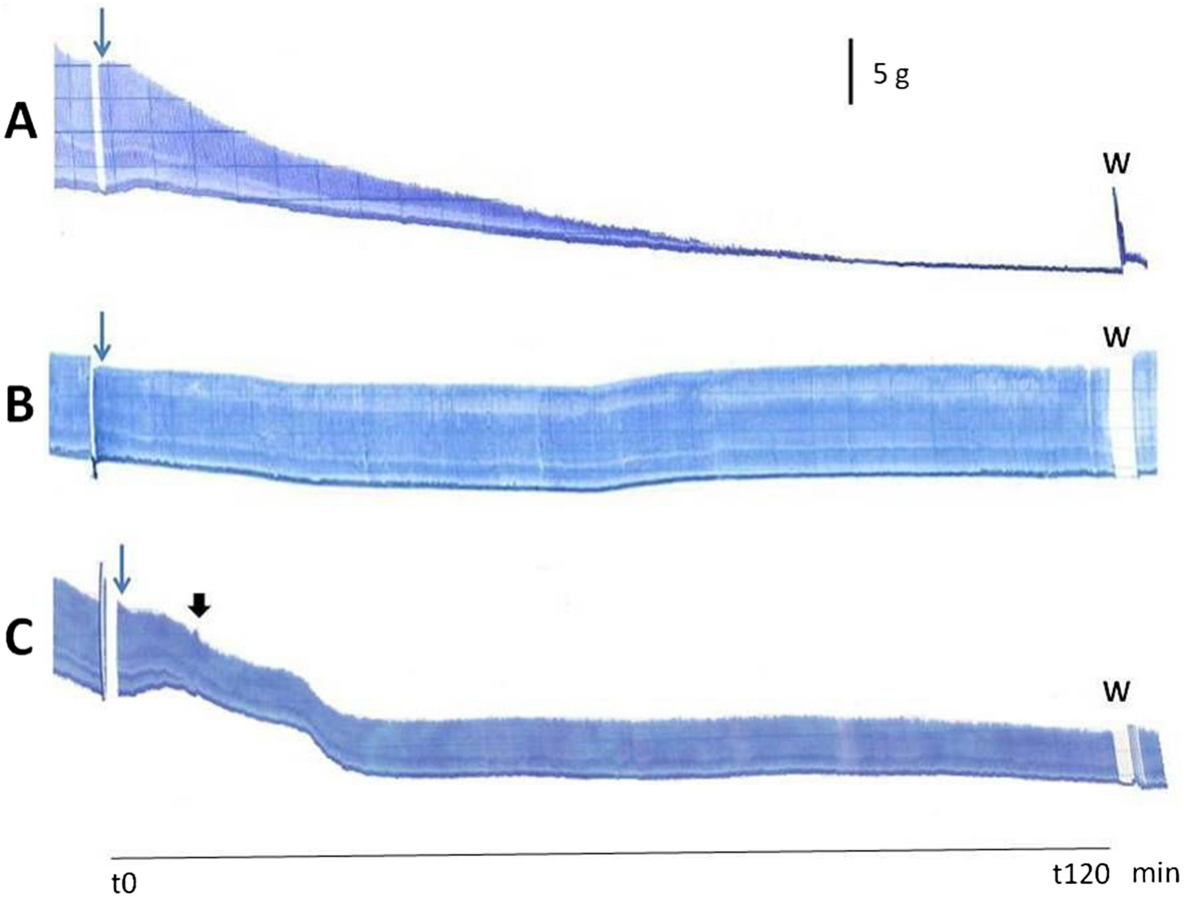
**Representative recordings of the responses to: A, *B. jararacussu *venom (40 μg/mL) showing its characteristic irreversible neuromuscular blockade (even after washing of preparation); B, pre-incubation of 40 μg/mL venom and 1 mg/mL extract during 30 min before addition into the bath; C, Extract addition (1 mg/mL, at 10 min, black arrow) in venom-pretreated preparation (40 μg/mL).** Blue arrows: start of experiment (zero time). Black arrow: extract addition. W = washing. Vertical bar = tension.

Figure 4 shows the representative myographic register of *B. jararacussu* venom (A, 40 μg/mL) exhibiting the characteristic irreversible muscle paralysis. When venom (40 μg/mL) + *V. flavicans* extract (1 mg/mL) are preincubated together for 30 min before addition into the bath, the toxic effect of the venom was not expressed (B). This result shows the total protection given by the plant extract against the venom, probably by a mechanism that excludes protein precipitation, as seen in tannins and tannic acid [45]. Even in preparations pretreated with *B. jararacussu* (40 μg/mL) for 10 min, which showed contracture and 40% of visible paralysis, the addition of 1 mg/mL *V. flavicans* extract into the bath was able to delay the paralysis progression, in a non-preincubated model (C). The mechanism of the *in vitro*, but not the *in vivo*, neuromuscular paralysis caused by *B. jararacussu* venom was recently demonstrated [36]. There is a direct relationship between the cell damage level and the percentage of twitch tension response [43].

The antimicrobial activities (MIC and MBC) of *V. flavicans* extract against *E. coli*, *E. faecalis*, *P. aeruginosa*, and *S. aureus* strains values are shown in Table 1. All susceptible bacterial species were inhibited or killed by vancomycin and/or gentamicin, in accordance to the recommendations of CLSI. Ethanol (extract solvent) did not interfere with bacterial growth in the concentrations tested.

**Table 1:** **Minimum inhibitory (MIC) and bactericidal (MBC) concentrations (in mg/mL) for *V. flavicans *** **extract against *E. coli*, *E. faecalis*, *P. aeruginosa *** **and *S. aureus *** **strains**

**Bacterial strains**	***V. flavicans***	**Vancomycin**	**Gentamicin**
	**MIC and MBC (mg/mL)**		
*E. coli* ATTC 25922	NF	NF	0.0008
*E. coli* ATTC 10536	NF	NF	0.0004
*E. faecalis* ATTC 29212	NF	0.0015	0.0125
*P. aeruginosa* ATTC 25619	2.5	NF	0.0002
*P. aeruginosa* ATTC 27853	2.5	NF	0.0004
*S. aureus* ATTC 6538	2.5	0.0008	0.0008
*S. aureus* ATTC 14458	2.5	0.0008	0.0004
*S. aureus* ATTC 29213	2.5	0.0008	0.0004
*S. aureus* ATTC 33591	2.5	0.0008	0.0015
*S. aureus* ATTC 43300	5.0	0.0008	NF

NF - no MIC or MBC were found considering the concentrations tested.

To assess the antimicrobial activity of a plant extract, strict endpoint criteria for MIC, IC_50_ and/or IC_90_ must be considered. Generally an extract can be considered effective if it shows IC_50_ lower than 100 μg/mL [47]. The MIC values found in the present study were up to 50 times higher than the concentration considered as the maximum concentration acceptable for IC_50_. Therefore, *V. flavicans* extract does not possess relevant antibacterial activity, considering the high values of MIC and MBC for *P. aeruginosa* and *S. aureus* strains (2.5–5.0 mg/ml) and the absence of inhibition of *E. coli* and *E. faecalis* strains.

Some specific plants extracts and metabolites can be effective against microorganisms and snake envenomation. *Andrographis paniculata* demonstrated activity against different pathogenic microorganisms [48] and it has anti-venom activity against *Naja naja* venom [49]. *Vitis vinifera* can effectively inhibit toxic effects, such as edema, hemorrhage, myonecrosis, and coagulation of human plasma induced by *Echis carinatus* venom [50]. *V. vinifera* can also inhibit the growth of *S. aureus*, *E. coli*, and *Bacillus cereus*[51]. A plant extract showing both anti-venom and antimicrobial activities would be very useful since it could be used to prevent or to treat local/systemic infection caused by snakebite [2]. Although *V. flavicans* extract did not show good antimicrobial activity and a more appropriate experimental inflammation model was not used [34], the extract showed potential for treating *Bothrops* snake envenomation.

## Conclusions

In conclusion, the *V. flavicans* hydroalcoholic extract showed promising results against the *in vitro* neuromuscular blockade-induced by *B. jararacussu* venom at the nerve-muscle apparatus. The extract reduced the characteristic cell damage induced by the venom and avoided cell damage progression.

## Abbreviations

ATCC: American Type Culture Collection; B. jararacussu: Bjssu, *Bothrops jararacussu*; CLSI: Clinical and Laboratory Standards Institute; CFU: Colony forming units; DMSO: Dimethyl sulfoxide; D. alata: *Dipteryx alata*; E. faecalis: *Enterococcus faecalis*; E. coli: *Escherichia coli*; MRSA: *M*ethicillin-resistant *S. aureus*; MBC: Minimum bactericidal concentration; MIC: Minimum inhibitory concentration; MHB: Mueller Hinton Broth; ORSA: Oxacillin resistant *S. aureus*; PEG: Polyethylene glycol; PND: Phrenic nerve diaphragm; P. aeruginosa: *Pseudomonas aeruginosa*; Rf: Retention factor; TLC: Thin Layer Chromatography; TSA: Tryptic soy agar; TSB: Tryptic soy broth; V. flavicans: Vf, *Vellozia flavicans*; S. aureus: *Staphylococcus aureus*.

## Competing interests

The authors declare that they have no competing interests.

## Authors’ contributions

The students NT, AMS and MCF were responsible for the pharmacological methodology under YOF advisory. MGS was responsible for plant quality control and chromatography. The students APGB and TSG carried out the antimicrobial assays under KC advisory. MGS was responsible for collection of plant samples obtained in Tocantins. JCC was responsible by venom certification. EAV collaborated during the manuscript writing. FCG was responsible for manuscript criticism and English language use and correction. All authors have read and approved the final manuscript.

## Pre-publication history

The pre-publication history for this paper can be accessed here:

http://www.biomedcentral.com/1472-6882/14/48/prepub
